# Efficiency improvement of AlGaInP-based red micron-scale light-emitting diodes using sidewall steam oxidation

**DOI:** 10.1186/s11671-025-04241-7

**Published:** 2025-03-26

**Authors:** Yuan-Chao Wang, Cheng-Jui Yu, Jian-Jang Huang

**Affiliations:** 1 Graduate Institute of Photonics and Optoelectronics, National Taiwan University, Taipei, 10617 Taiwan; 2 Department of Electrical Engineering, National Taiwan University, Taipei, 10617 Taiwan

**Keywords:** Micro-LEDs, AlGaInP, Sidewall steam oxidation, Luminous efficacy, Surface passivation

## Abstract

Although micro-LED displays are considered emerging display technology, their micron-scale LED chip size suffers from significant efficiency degradation, which affects the display's power budget. The low light output efficiency is mainly attributed to an increased weighting of sidewall nonradiative recombination with the perimeter-area ratio of smaller chip size. To prevent carrier recombination in the dry-etching induced sidewall defects, we, in this study, introduce insulting regions in the mesa sidewall of the red LED. The insulting regions were created by oxidizing the metal components in the epi-structures. When the chip sizes of 100 × 100, 50 × 50, and 25 × 25 μm^2^ are compared, our steam oxidation technique efficiently suppresses sidewall current flow and nonradiative recombination. The suppression is more obvious for a smaller mesa size. For a 25 × 25 μm^2^ LED mesa, optical output power density increases by 31.4% compared to a device without oxidation. Additionally, under 20 A/cm^2^ injection, a 25 × 25 μm^2^ LED with sidewall oxidation shows only an 11.3% reduction in output power density compared to a larger 100 × 100 μm^2^ device without oxidation. These results highlight the potential of sidewall oxidation in overcoming efficiency degradation issues for micro-red LEDs in displays.

## Introduction

Micro-LED (light-emitting diode) (µLED) displays have the advantages of a high-contrast ratio, low response time, flexible panel form factor, and potentially low power consumption [1–5]. Though tremendous efforts have been made to drive the technology into commercialization in recent years [6, 7], there are still many challenges. For example, µLED displays require a micron-scale LED chip size for mass transfer [8, 9]. However, the LEDs' external quantum efficiency (EQE) decreases significantly with the mesa size shrink due to a more pronounced sidewall nonradiative recombination [10–12]. For nitride-based LEDs, the typical strategy is to increase light output efficiency through material design [5, 13], sidewall treatment [10, 14], or optimized die-mount technology [6, 15]. The situation is even worse for red LEDs because the AlGaInP-based epistructure possesses a surface recombination velocity an order of magnitude higher than the green and blue counterparts that employ III-nitride materials [16–19]. For example, when the size of AlGaInP red LEDs is reduced from 100 × 100 to 20 × 20 µm^2^, the relative EQE (external quantum efficiency) decreases by approximately 80% at 20 A/cm^2^ current density, estimated from [20]. In contrast, for GaN-based blue LEDs, the relative EQE decreases by less than 30% (estimated from [21]), giving the same size reduction and current density.

Researchers have explored various techniques to improve the EQE of small red LEDs. The most common approaches are to passivate sidewall defects or to remove the damaged sidewalls. For example, after dry etching, the GaAs-based µLEDs are treated with ammonium sulfide [22] or Citric Acid [23]. Sidewall passivation was also carried out by various dielectric deposition methods [20, 24, 25]. Since the metal composition in the AlGaInP material layers can be oxidized with proper treatment to form metal-oxide sidewall-insulated regions [26, 27], we proposed sidewall stream oxidation to prevent current flow across the dry-etching-induced damaged region in this study. We demonstrated a notable improvement in the optical properties of AlGaInP µLEDs and the suppression of reverse leakage current in the diodes. Our study addresses surface recombination challenges in AlGaInP µLEDs, paving the way for highly efficient red AlGaInP µLEDs suitable for display applications.

## Device fabrication

We employed metal–organic chemical vapor deposition (MOCVD) for red LED epi-growth on the n-type GaAs substrate. The LED epitaxial structure consists of a 200 nm-thick n-type GaP layer, a 30 nm n-type GaAs layer, a 320 nm n-type AlGaInP cladding layer, 3 periods of AlGaInP/GaInP multiple quantum wells (MQWs) with an EL (electroluminescent) emission peak wavelength of 622 nm, a 280 nm p-type AlGaInP cladding layer, and a 4000 nm p-type GaP contact layer. We defined the light-emitting mesa area by inductively coupled plasma reactive ion etching (ICP-RIE) with an etching depth of 5200 nm to expose the n-type cladding layer. The remaining n-type cladding layer was removed by wet etching in a H_3_PO_4_: HCl (3:1) solution.

In this study, we fabricated LEDs with three mesa sizes: 25 ×25, 50 × 50, and 100 × 100 μm^2^. The samples were next placed in the furnace to oxidize the mesa sidewall with the process condition described in the next paragraph. Before metal deposition, a low-power Ar bombardment using RIE (reactive ion etching) was performed to remove surface oxide after photolithography. We deposited p-type contact metal (Ti/Au 15/120 nm) by e-beam evaporation followed by rapid thermal annealing (RTA) at 650 °C for 100 s in N_2_ ambient to form an ohmic contact. A Ni/Ge/Ni/Au (5/15/50/400 nm) metal stack was evaporated as the n-type contact, followed by 410 °C alloying for 60 s in N_2_ ambient. We then utilized plasma-enhanced chemical vapor deposition (PECVD) to deposit a 100 nm-thick SiN dielectric layer to further passivate the device. Finally, we employed RIE to open contact VIAs. For comparisons, we fabricated devices without sidewall oxidation, but the rest of the process steps were the same.

**Fig. 1:**
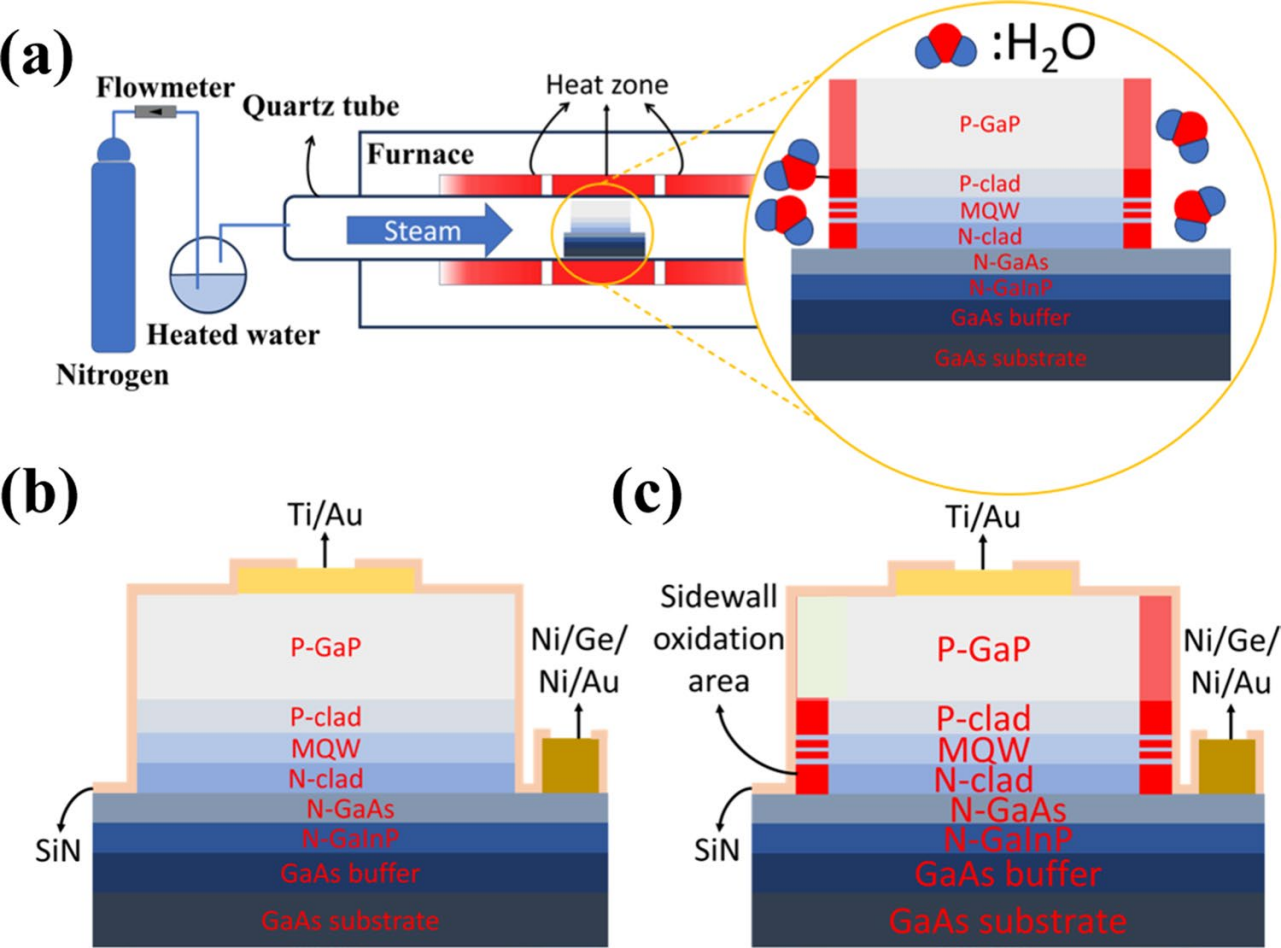
**a** Illustration of the wet oxidation process in the furnace tube. Side view profile of the micro-LED **b** without sidewall oxidation and **c** with sidewall oxidation

**Fig. 2:**
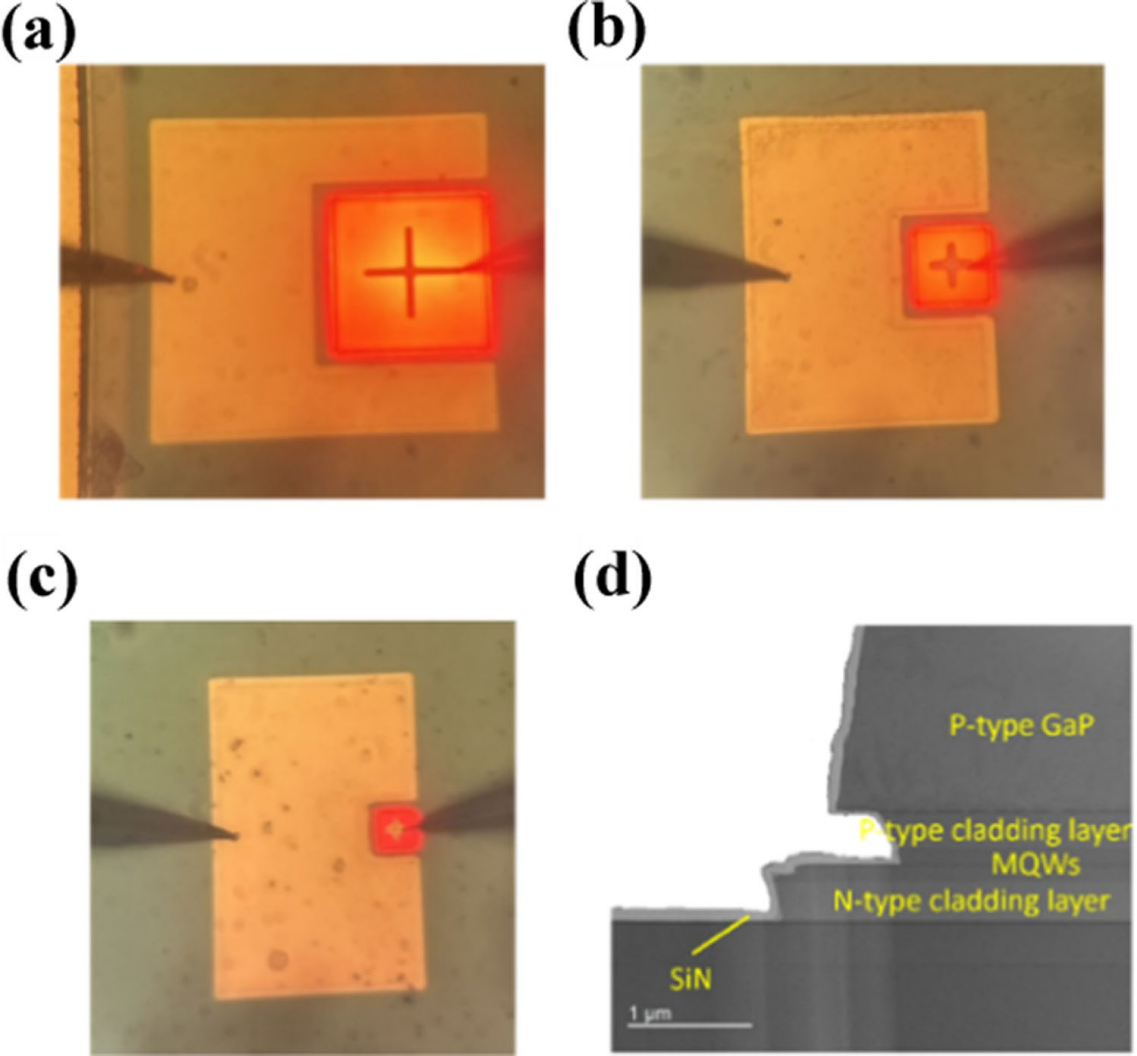
Luminescence from the device **a** B-100, **b** B-50, and **c** B-25. The current density is 20 A/cm^2^. **d** TEM image of mesa sidewall of B-25

Figure 1a shows the wet oxidation process in the furnace tube. The samples were first purged with N_2_ gas flow at 300 °C for 5 min before oxidizing in steam (water vapor) under three oxidation conditions as listed in Table 1. The lateral epi-structural profile of the devices without sidewall oxidation is shown in Fig. 1b. The Al component is oxidized during oxidation, converting the mesa sidewall to Aluminum Oxide, as shown in red in Fig. 1c. In this work, the nomenclature of devices follows the “sidewall oxidation scheme-LED mesa length” format. For example, A-100 means an LED with a mesa size of 100 × 100 μm^2^ using the oxidation Scheme A. Additionally, N-25, N-50, and N-100 indicate LEDs without sidewall oxidation. Figure 2a, b, and c show the luminescence of devices B-100, B-50, and B-25, respectively. The TEM (Transmission electron microscopic) image of the B-25 side view is shown in Fig. 2d. Because of the etching rate difference between the p-type GaP, cladding layer, and MQWs, we observed an undercut in the p-type cladding layer.

**Table 1:** Process parameters for sidewall oxidation

Oxidation scheme	Oxidation conditions
N	Without oxidation (Reference)
A	300 °C 30 min
B	300 °C 60 min
C	300 °C 90 min
D	350 °C 60 min

## Results and discussion

**Fig. 3:**
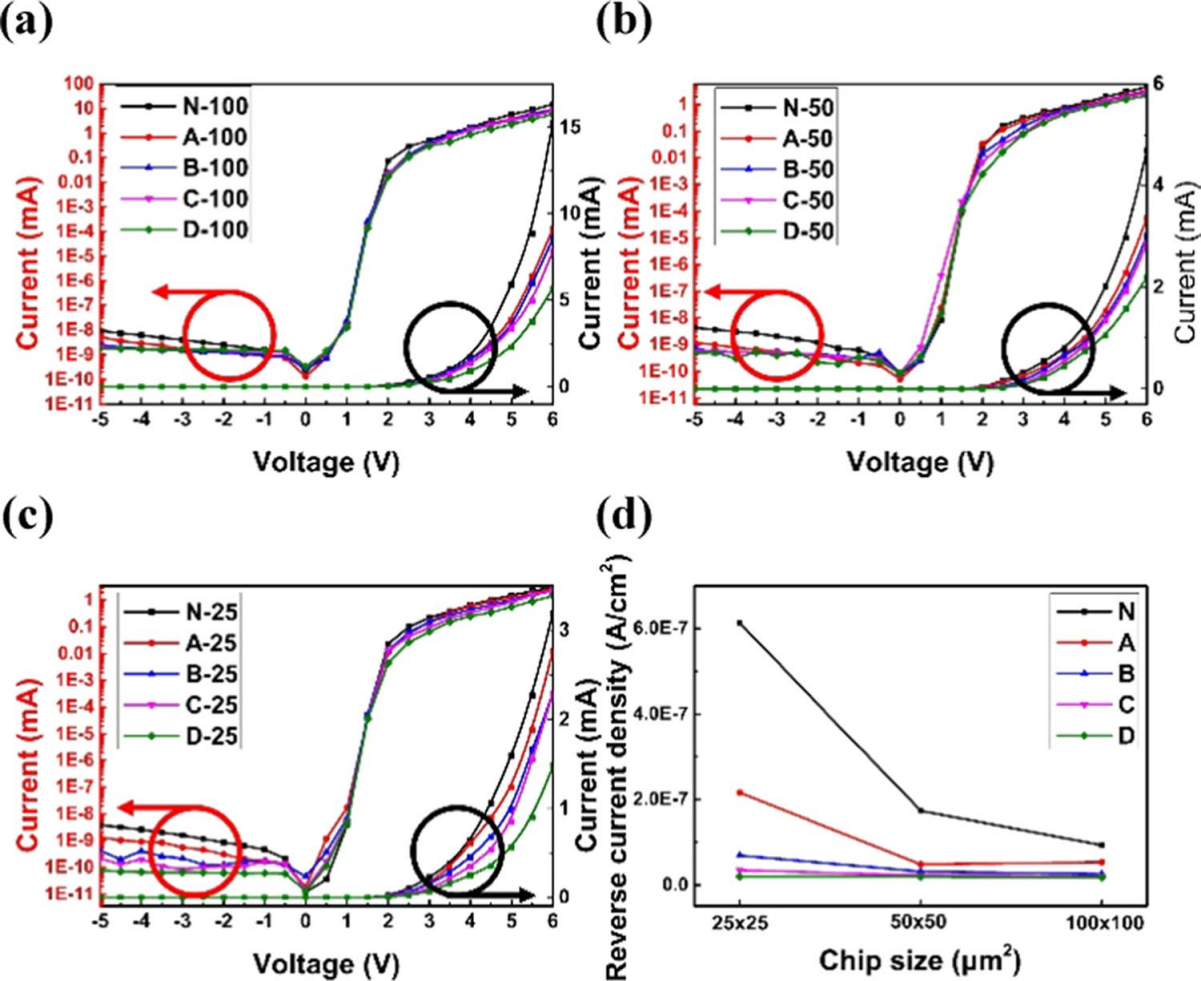
I–V curves of LEDs with the mesa size of **a** 100 × 100 μm^2^, **b** 50 × 50 μm^2^, and **c** 25 × 25 μm^2^. **d** Comparisons of the devices' reverse current densities at − 5 V

We first characterized the electrical properties of the devices using an Agilent 4155C semiconductor parameter analyzer. Figure 3a, b, and c show the injection current–voltage (I–V) curves of LEDs. For all three mesa sizes, the sidewall oxidized devices' forward currents are lower than those without oxidation, which is mainly attributed to a reduced effective mesa area with oxidation. The forward current decreases in the order N, A, B, C, and D. It implies that the oxidized region is widened when the oxidation time and temperature increase. Moreover, oxidation Scheme D has the lowest forward current among the schemes, suggesting that temperature plays a critical role in the oxidation process, which will be further verified in the following study.

We also observe a strong correlation between sidewall leakage currents and the range of oxidation. To understand the effect of sidewall oxidation, the reverse current densities, i.e., currents normalized to each mesa size, at −5 V of the devices are plotted in Fig. 3d. When the reverse current density at a specific mesa size is compared, it decreases in the order of oxidation Scheme N, A, B, C, and D, a trend similar to that of the forward current. It indicates that sidewall leakage can be effectively suppressed with oxidation. The leakage depends on the range of oxidation. Another reason for suppressed leakage current density is the decrease in effective mesa area. Furthermore, we also compare the current densities when the mesa size shrinks from 100 × 100 to 25 × 25 μm^2^. Without oxidation, the leakage current density increases by 6.6 times because the surface leakage across the sidewall gradually prevails. For oxidation Schemes C and D, the leakage current only increases by 1.7 and 1.6 times, respectively. The slight increase in reverse current density suggests that sidewall oxidation effectively suppresses sidewall surface current flow.

**Fig. 4:**
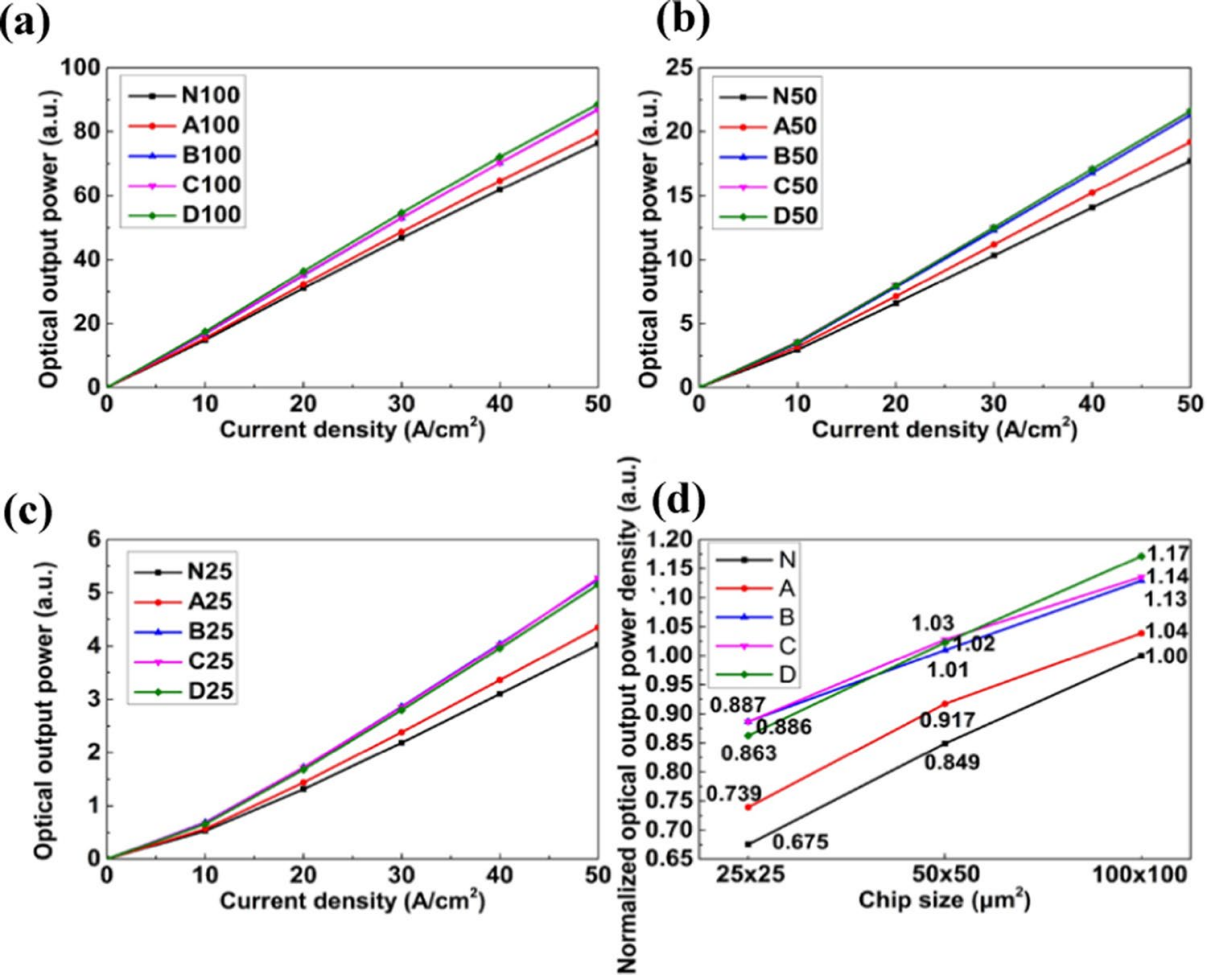
L-J curves of LEDs with a mesa size of **a** 100 × 100 μm^2^, **b** 50 × 50 μm^2^, and **c** 25 × 25 μm^2^. **d** Normalized optical output power density of LEDs

Next, we examined the emission characteristics using the Ophir StarBright photonic power meter. Figure 4a, b, and c shows the optical output power versus injection current density (L–J) curves of LEDs with sizes of 10 × 0 × 100, 50 × 50, and 25 × 25 μm^2^. 5 devices of each type distributed at various locations of the 1 × 1 cm^2^ sample were measured. From the L-J curves, sidewall oxidation effectively increases light output, suggesting the suppression of nonradiative recombination in the mesa periphery. Light output improvement is nearly saturated when the oxidation time exceeds 60 min at 300 °C. Also, as we further increase the temperature to 350 °C, the oxidation condition may not be the optimum parameter for light output. For a larger mesa size of 100 × 100 μm^2^, the light output of D-100 is the highest among the oxidation schemes. However, when we shrink the mesa size to 25 × 25 μm^2^, the light output of B-25 and C-25 is higher than D-25. It implies the effective mesa area becomes too small to compensate for suppressing sidewall nonradiative loss.

The above results reinforce the proposed idea that sidewall oxidation plays a role in improving optical output. The enhanced optical output is attributed to the reduced sidewall surface defect states and the mitigation of sidewall leakage current. In Fig. 4d, all the devices' output power density is normalized to N-100, the largest device without sidewall oxidation. Compared to N-100, A-100, B-100, C-100, and D-100 show enhancements of average output power by 3.8, 12.8, 13.5, and 17.1%, respectively. When the mesa size shrinks to 25 × 25 μm^2^, B-25 is brighter than N-25 by 31.4%. It indicates that the smaller size (25 × 25 μm^2^) improvement is more significant than that in large size (100 × 100 μm^2^). When the size degradation effect is evaluated, the output power density of B-25 is degraded by 11.3%, compared with N-100. Again, it suggests that sidewall oxidation can effectively suppress the efficiency drop at a smaller LED size.

**Fig. 5:**
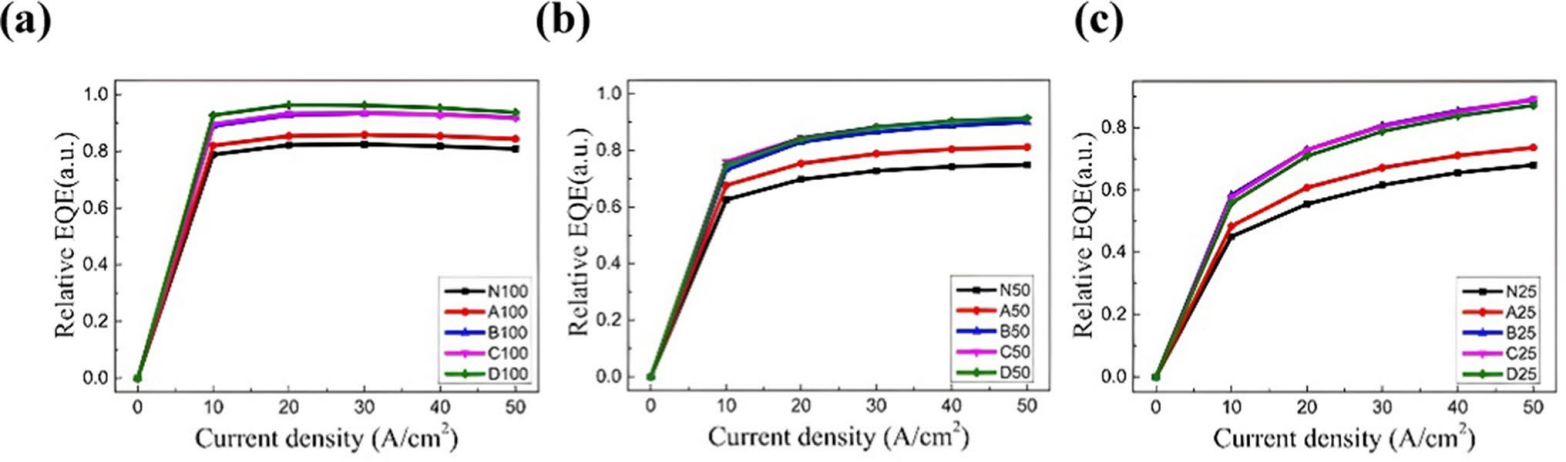
Relative EQE characteristics of **a** 100 × 100 μm^2^, **b** 50 × 50 μm^2^ **c**) 25 × 25 μm^2^ LEDs

Figure 5 illustrates the current density dependence of relative EQE of 100 × 100, 50 × 50, and 25 × 25 μm^2^ devices. Among the current density measurement range, 100 × 100 and 50 × 50 μm^2^ devices with oxidation scheme D possess higher maximum relative EQE (EQE_max_) than other oxidation conditions, while scheme C has higher EQE_max_ for 25 × 25 μm^2^ devices. Furthermore, the current density that reaches EQE_max_, I_peak_, is smallest for 100 × 100 μm^2^ devices, while the relative EQE is not saturated yet for the injection current density up to 50 A/cm^2^. Following the ABC model, a higher I_peak_ suggests a higher A/B ratio [28]. It indicates a higher Shockley–Read–Hall (SRH) recombination for smaller devices. With the sidewall oxidation, the relative EQE is improved by suppressing Shockley–Read–Hall (SRH) recombination.

**Fig. 6:**
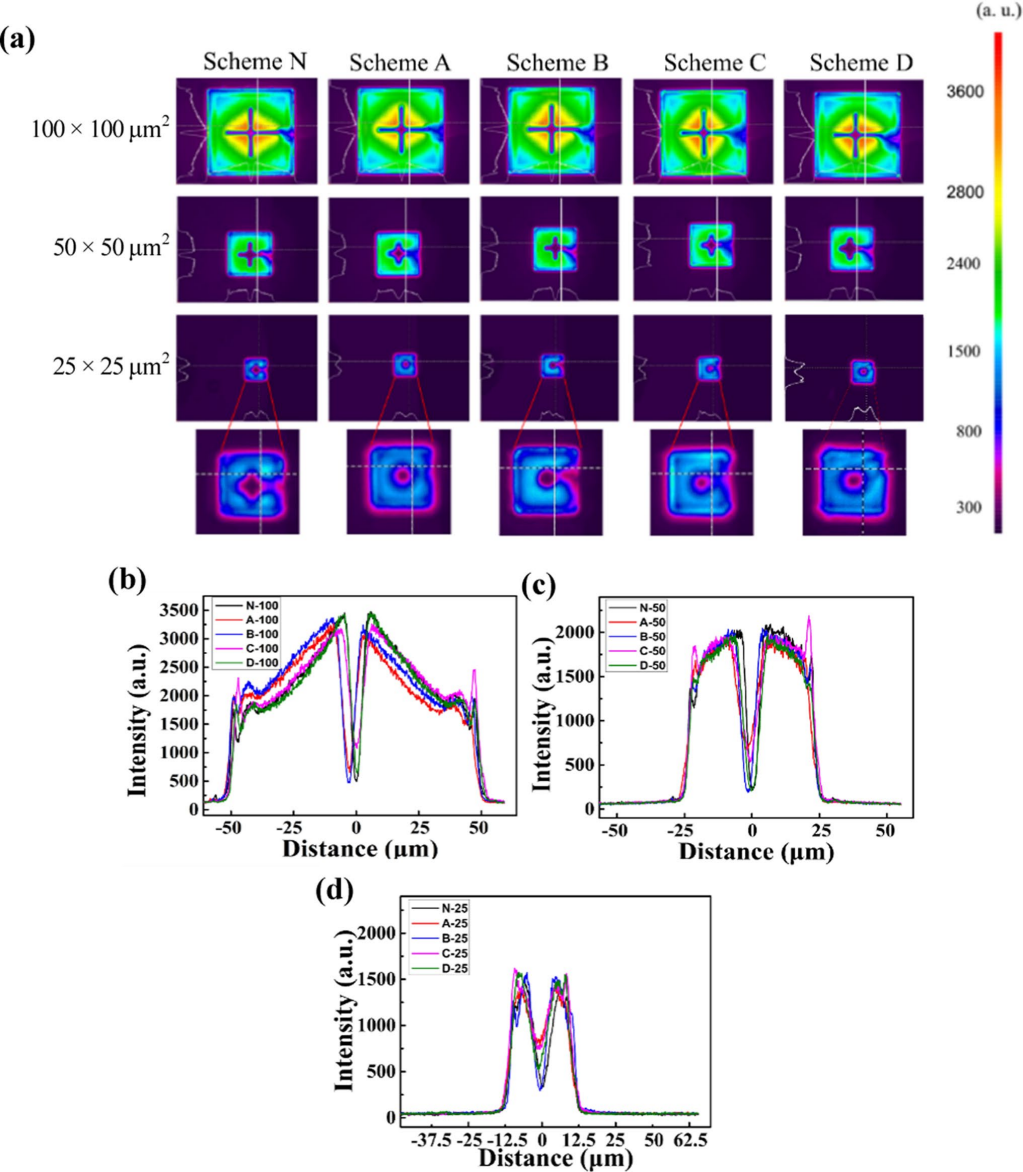
**a** EL images of the LEDs. Moreover, comparisons of light emission profiles across the vertical white lines labeled in the images of LEDs with mesa size of **b** 100 × 100 μm^2^, **c** 50 × 50 μm^2^, and **d** 25 × 25 μm^2^

Next, we captured EL images to analyze LED light emission distribution. Figure 6a shows the images of all the devices investigated in the work. The inject current density is 20 A/cm^2^. Also, the light extraction software Beammic was employed to numerically compare light emission profiles among devices. Figure 6b, c, and d compare the emission intensities along the y-axis (white lines in Fig. 6(a)) of the devices with mesa sizes 100 × 100, 50 × 50, and 25 × 25 μm^2^, respectively. The light output decreases from the center toward the mesa edges for all LED sizes. Light output at the edges will increase because of lateral light propagation and extraction from the MQW sidewall edge. However, with a proper sidewall oxidation process, as demonstrated by LEDs using Scheme B and C, we observe brighter light emission at the edges than the device without oxidation. Moreover, compared with Scheme N, edge light output improvement is more pronounced for smaller device sizes. The emission profile analysis agrees with our observation in Fig. 4 that proper sidewall oxidation effectively enhances the light distribution. Moreover, our assumption on deteriorated sidewall emission using Scheme D is verified from the EL images. Devices with all three different sizes show dimmer light output on the edges using Scheme D, compared with the rest of the oxidation parameters.

**Fig. 7:**
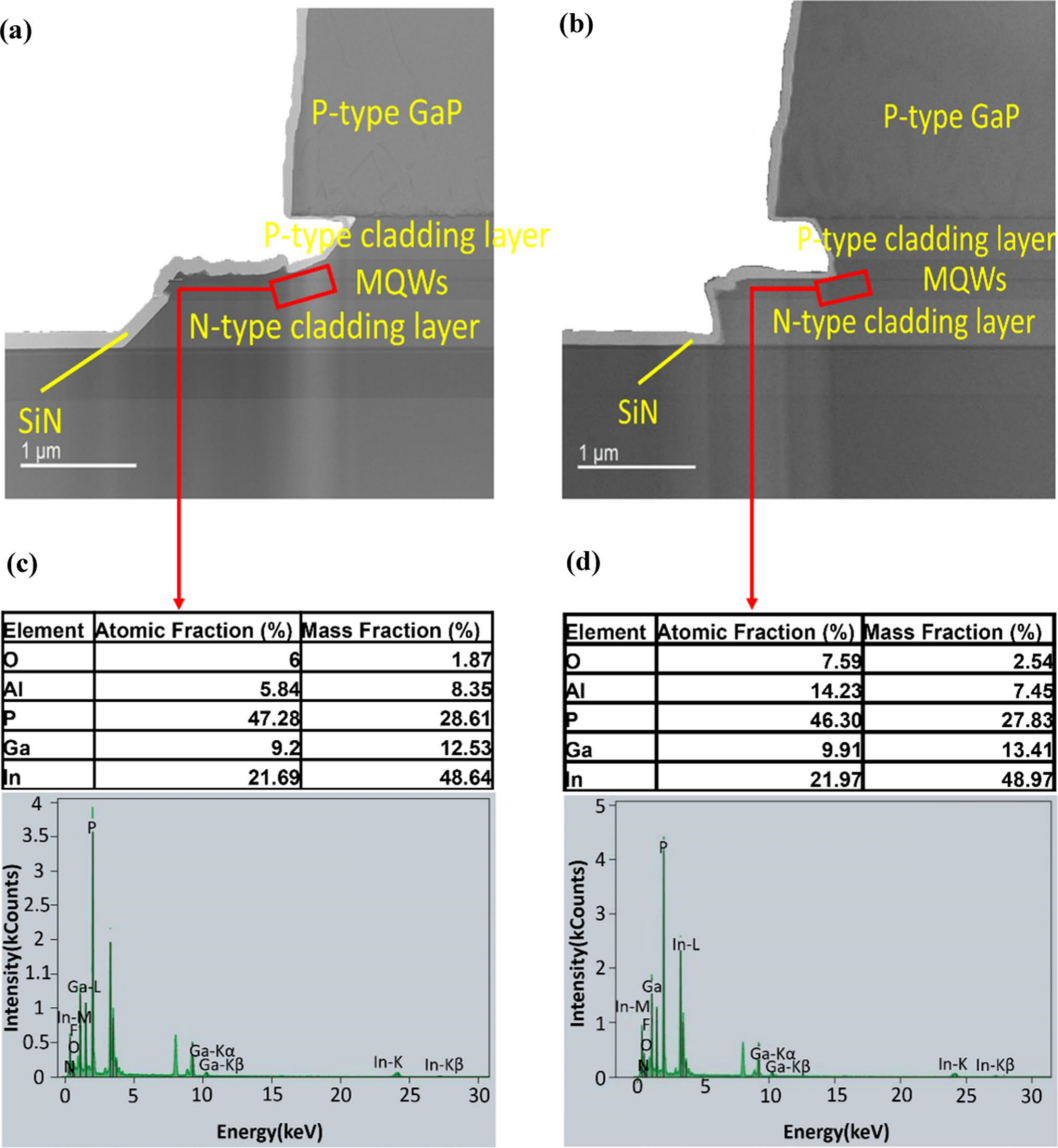
TEM image of LEDs **a** without sidewall oxidation **b** with the oxidation Scheme B. EDX spectrum of MQW edges **c** without sidewall oxidation **d** with the oxidation Scheme B. EDX mapping of oxygen **e** without sidewall oxidation **f** with the oxidation Scheme B

Next, we performed an energy-dispersive X-ray spectroscopy (EDX) analysis on a sample with Scheme B. Another sample without oxidation, Scheme N, is compared. The TEM images of the sidewalls with Scheme N and B are shown in Fig. 7a and b, respectively. The element distribution in the MQWs layer, shown in Fig. 7c and d, suggests the oxygen atomic fraction increases from 6.00% of Scheme N to 7.59% of Scheme B. The above observations confirm that the sidewall oxidation process successfully oxidized the material on the sidewalls, avoiding the sidewall current flow toward the MQWs.

**Fig. 8:**
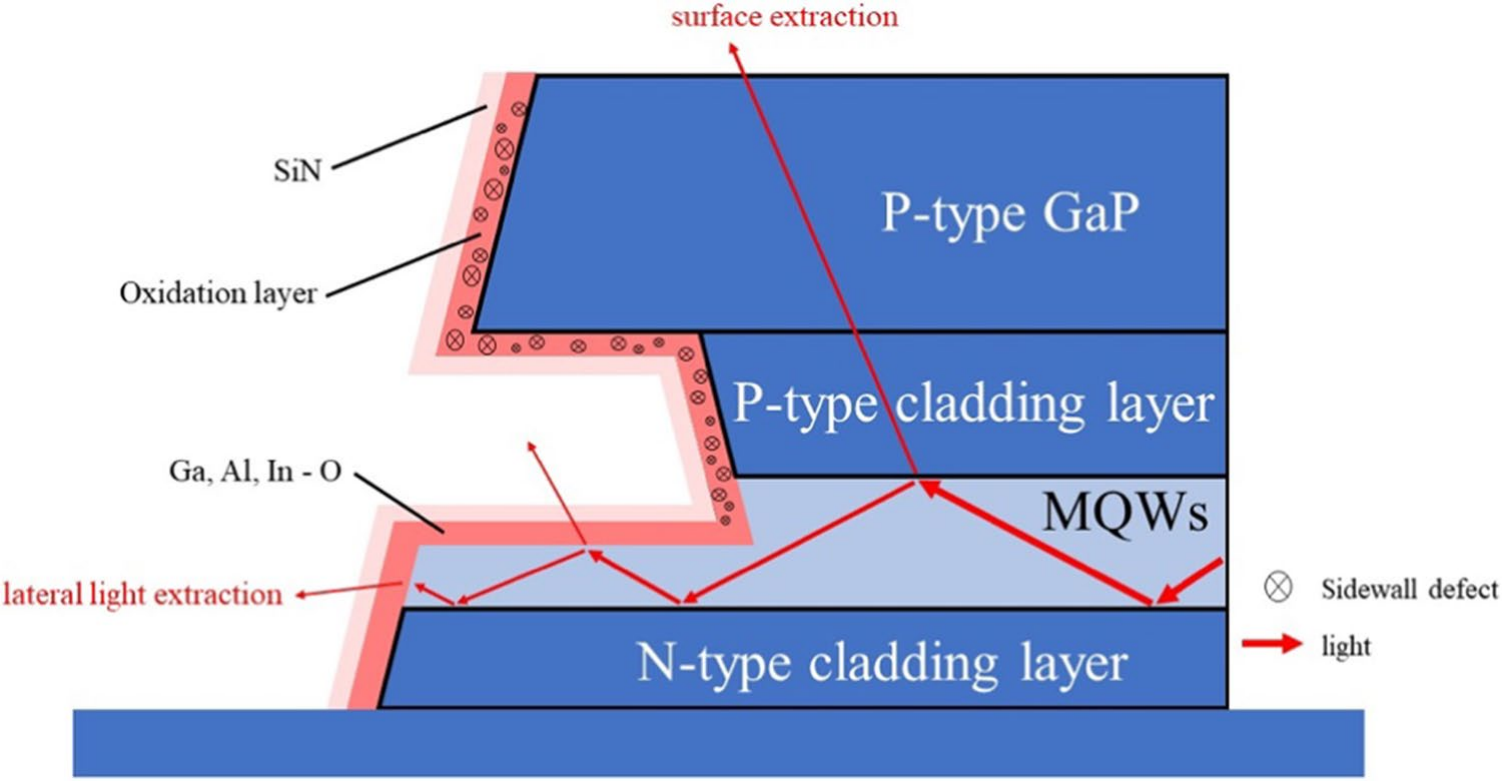
Illustration of light extractions from the surface and lateral direction. With oxidized sidewall, sidewall surface current is suppressed, and the graded refractive index change enhances lateral light extraction

From the TEM images and device properties exhibited above, the light output enhancement with the sidewall oxidation can be attributed to the following mechanisms. As shown in Fig. 8, the injection current flowing across the sidewall is suppressed because of the oxidized insulating layer in the sidewalls. The nonradiative recombination of carriers with dry-etching-induced defects is reduced. Second, lateral light output is enhanced due to low-refractive index metal oxide formation in the sidewalls. The smaller refractive index of metal oxide (than those of the epilayers) creates a refractive index graded change from the semiconductor, metal oxide, and SiN_x_ to the air when light is extracted laterally from the sidewalls.

## Conclusion

We demonstrate the sidewall oxidation technique to effectively mitigate sidewall carrier leakage and nonradiative recombination, thereby enhancing the optical properties of micro-LEDs. Specifically, for micro-LEDs with small chip sizes, the output power of B-25 is 31.4% higher than N-25. Moreover, when the impact of size-related efficiency decrease is considered, the output power density of B-25 only degrades by 11.3%, compared with N-100. The proposed sidewall oxidation technique shows great potential for enhancing micro-LED performance and advancing their applications in high-performance displays and optoelectronic devices.

## Data Availability

No datasets were generated or analysed during the current study.
